# miR‐200b ameliorates myofibroblast transdifferentiation in precancerous oral submucous fibrosis through targeting ZEB2

**DOI:** 10.1111/jcmm.13690

**Published:** 2018-06-12

**Authors:** Yi‐Wen Liao, Cheng‐Chia Yu, Pei‐Ling Hsieh, Yu‐Chao Chang

**Affiliations:** ^1^ School of Dentistry Chung Shan Medical University Taichung Taiwan; ^2^ Department of Dentistry Chung Shan Medical University Hospital Taichung Taiwan; ^3^ Institute of Oral Sciences Chung Shan Medical University Taichung Taiwan

**Keywords:** miR‐200b, myofibroblast, oral submucous fibrosis, ZEB2

## Abstract

Oral submucous fibrosis (OSF) is a progressive scarring disease. MicroRNA‐200b (miR‐200b) has been reported as a tumour suppressor, but its role in the precancerous OSF remains unknown. In this study, we investigated the impact of miR‐200b on myofibroblastic differentiation activity. Arecoline is a major areca nut alkaloid and has been employed to induce the elevated myofibroblast activity in human buccal mucosal fibroblasts (BMFs). Treatment of arecoline in BMFs dose‐dependently reduced gene expression of miR‐200b, which corresponded with the decreased expression of miR‐200b in fBMFs. The arecoline‐induced myofibroblast activities were abolished by overexpression of miR‐200b in BMFs, and the same results were observed in fBMFs. In addition, α‐SMA was inhibited by an increase in miR‐200b. We further demonstrated that miR‐200b‐mediated decrease in ZEB2 led to down‐regulation of α‐SMA, vimentin. Loss of miR‐200b resulted in enhanced collagen contraction and migration capabilities, and knockdown of ZEB2 reversed these phenomena. Lastly, we showed the expression of miR‐200b was significantly less and ZEB2 was markedly higher in OSF tissues. These results suggested that down‐regulation of miR‐200b may contribute to the pathogenesis of areca quid‐associated OSF through the regulation of ZEB2 and myofibroblast hallmarks.

## 1 INTRODUCTION

Oral submucous fibrosis (OSF) is a chronic progressive scarring disease and has been recognized as one of the oral potentially malignant disorders. This precancerous condition of the oral mucosa is associated with inflammatory cell infiltration followed by the accumulation of connective tissue in lamina propria and epithelial atrophy,^1^, ^2^ leading to difficulty of mouth opening. Epidemiological evidence has attributed areca quid chewing habit as the main aetiological factor for OSF.^3^, ^4^ However, the precise pathogenesis of OSF still remains elusive and effective treatment is lacking.

Myofibroblasts, the α‐smooth muscle actin (SMA)‐expressing contractile fibroblasts,^5^ play a critical role in wound healing and tissue remodelling.^6^ However, persistent activation of myofibroblasts often leads to pathological fibrosis as it has been implicated in the dysregulation of extracellular matrix (ECM) synthesis and degradation.^6^, ^7^ Myofibroblast accumulation indeed has been found in multiple tissue fibroses, including OSF.^8^ Among multiple sources of myofibroblast precursors, it has been indicated that epithelial‐mesenchymal transition (EMT) contributes to tissue fibroses.^6^, ^7^, ^9^ EMT is a process in which epithelial cells transdifferentiate into motile mesenchymal cells, and it is mediated by various key transcription factors, including Snail (SNAI1), Slug (SNAI2), zinc‐finger E‐box‐binding (ZEB) and other basic helix‐loop‐helix transcription factors.^10^, ^11^ Also, intermediate filaments, such as vimentin, play a critical role in the induction of mesenchymal cell shape and motile behaviour.^12^ Our previous findings have shown that arecoline‐induced myofibroblast transdifferentiation is mediated by ZEB1,^13^ and inhibition of EMT transcription factor suppressed the pathogenesis of areca quid‐induced OSF through down‐regulation of myofibroblast activity.^14^ Hence, approaches to decrease the EMT regulating factors may be a promising strategy to inhibit the activation of myofibroblasts after arecoline stimulation, therefore preventing OSF pathogenesis.

MicroRNAs (miRNAs) are small, endogenous non‐coding RNAs of 21‐25 nucleotides in length that mediate the post‐transcriptional regulation of gene expression through partial complementary binding to the 3′untranslated region (UTR) in target transcripts.^15^, ^16^ Aberrant expression of miRNAs in fibrotic diseases has been reported in various studies (see Review^17^), and numerous miRNAs have been suggested to modulate EMT process, thereby contributing or inhibiting tissue fibroses.^18^, ^19^, ^20^ For instance, the EMT‐inducing transcriptional factors ZEB1 has been found to be a target of miRNA‐192 and inhibition of miR‐192 resulted in decreased collagen expression as well as reduced renal fibrosis.^21^ In addition, accumulating studies have demonstrated that miR‐200 family reduces fibrosis by inhibiting EMT and preventing the deposition of ECM.^22^, ^23^, ^24^ Also, it has been revealed that miR‐200b is associated with cancer metastasis,^25^ chemoresistance^26^ and prognosis.^27^ Hence, the impact of miR‐200b on precancerous OSF needs to be clarified.

In this study, we explored the functional role of miR‐200b in the pathogenesis of areca quid‐associated OSF and the associated mechanism. We tested the expression of miR‐200b in fibrotic cells and tissues, and investigated its contribution in arecoline‐induced and fibrotic myofibroblast activity, including higher collagen gel contractility and migration ability. Moreover, we assessed the expression of EMT‐associated factors in accordance with overexpression of miR‐200b and examined the relationship between miR‐200b and ZEB2 in BMFs. Our data revealed the anti‐fibrotic potential of miR‐200b in preventing the progression of OSF.

## 2 MATERIALS AND METHODS

### 2.1 Chemicals and cell culture

Arecoline and collagen solution from bovine skin were purchased from Sigma‐Aldrich (St. Louis, MO, USA). TGFβ type I receptor inhibitor, SB431542, was purchased from EMD Millipore (Billerica, MA, USA). Human TGFβ1 was obtained from R&D Systems (Minneapolis, MN, USA). All methods applied in this study were carried out in accordance with the approved guidelines from the Institutional Review Board of Chung Shan Medical University Hospital and informed written consent was obtained from each individual prior to commencing the study. Tissue specimens from areca quid chewers were collected from Department of Dentistry, Chung Shan Medical University Hospital, Taichung, Taiwan. Biopsy specimens were taken from the histologically normal oral mucosa and fibrotic mucosa at the time of surgical third molar extraction. Fibroblast cultures were grown and maintained by using the explant method as described previously.^28^ Cell cultures between the third and eighth passages were used in this study.

### 2.2 Collagen contraction assay

2 × 10^5^ BMFs were suspended in 0.5 mL of 2 mg/mL collagen solution (Sigma‐Aldrich) and then added into 24‐well plate followed by incubation at 37°C for 2 hours for the polymerization of collagen cell gels. After detaching gels from wells, the gels were further incubated in 0.5 mL MEMα medium with or without arecoline for 48 hours. Contraction of the gels was photographed and measured using ImageJ software (NIH) to calculate their areas.^29^

### 2.3 Migration assay

Cell migration assay was conducted using 24‐well plate Transwell^®^ system with a polycarbonate filter membrane of 8‐μm pore size (Corning, Acton, MA, USA) assay kit as previously described.^30^ The migrated cancer cells were then visualized and counted from 5 randomly selected fields under 100‐fold magnification using an inverted microscope.

### 2.4 Wound healing assay

Cells were seeded into 6‐well culture dishes. Wounds were introduced to the confluent monolayer of cells with a sterile 200 μL plastic pipette tip to create a denuded area. Cell movement towards the centre of the wound area was photographed at 0 and 48 hours under a microscope.^14^

### 2.5 Western blot analysis

Western blot analysis was conducted as previously described.^30^ The primary antibodies against α‐SMA, ZEB2, vimentin and Slug were purchased from Santa Cruz Biotechnology, Inc. (Santa Cruz, CA, USA). Following primary antibodies, the membranes were incubated with corresponding secondary antibodies. The immunoreactive bands were developed using an ECL‐plus chemiluminescence substrate (Perkin‐Elmer, Waltham, MA, USA) and captured by LAS‐1000 plus Luminescent Image Analyzer (GE Healthcare, Piscataway, NJ, USA).

### 2.6 Transfection of miRNA‐200b, ZEB2 3′UTR Reporter

Cells were seeded in 12‐well plate for attachment overnight and miRNA‐200b was transfected by LipofectamineTM 3000 transfection reagent (Invitrogen, Thermo Fisher Scientific Inc., Carlsbad, CA, USA) at a concentration of 100 nmol/L according to the manufacturer's protocol. A firefly luciferase reporter plasmid with full‐length ZEB2 3′‐UTR sequence was purchased from OriGene Technologies, Inc. (Rockville, MD, USA). The mutant ZEB2 3′‐UTR reporter plasmid used for deletion of the potential miRNA‐200b binding region (Figure 4A) was further constructed by QuickChange II XL Site‐Directed Mutagenesis Kit (Agilent Technologies Inc., Santa Clara, CA, USA). MiR‐200b overexpression plasmid construct was generated according to our previous method.

### 2.7 Apoptotic assay

Apoptotic cells were detected with an Annexin V‐APC kit (Calbiochem, Darmstadt, Germany) according to manufacturer's guidelines. After staining, the cells incubated with 20 μg/mL propidium iodide (PI) were analysed by FACS Calibur apparatus (Becton Dickinson, San Diego, CA, USA).

### 2.8 Statistical analysis

Quantitative data were presented as mean ± SD and analysed with Student's *t *test performed with SPSS Statistics version 13.0. *P* value < .05 was considered as statistically significant.

## 3 RESULTS

### 3.1 Mir‐200b is significantly down‐regulated in arecoline‐stimulated BMFs and fBMFs

**Figure 1:**
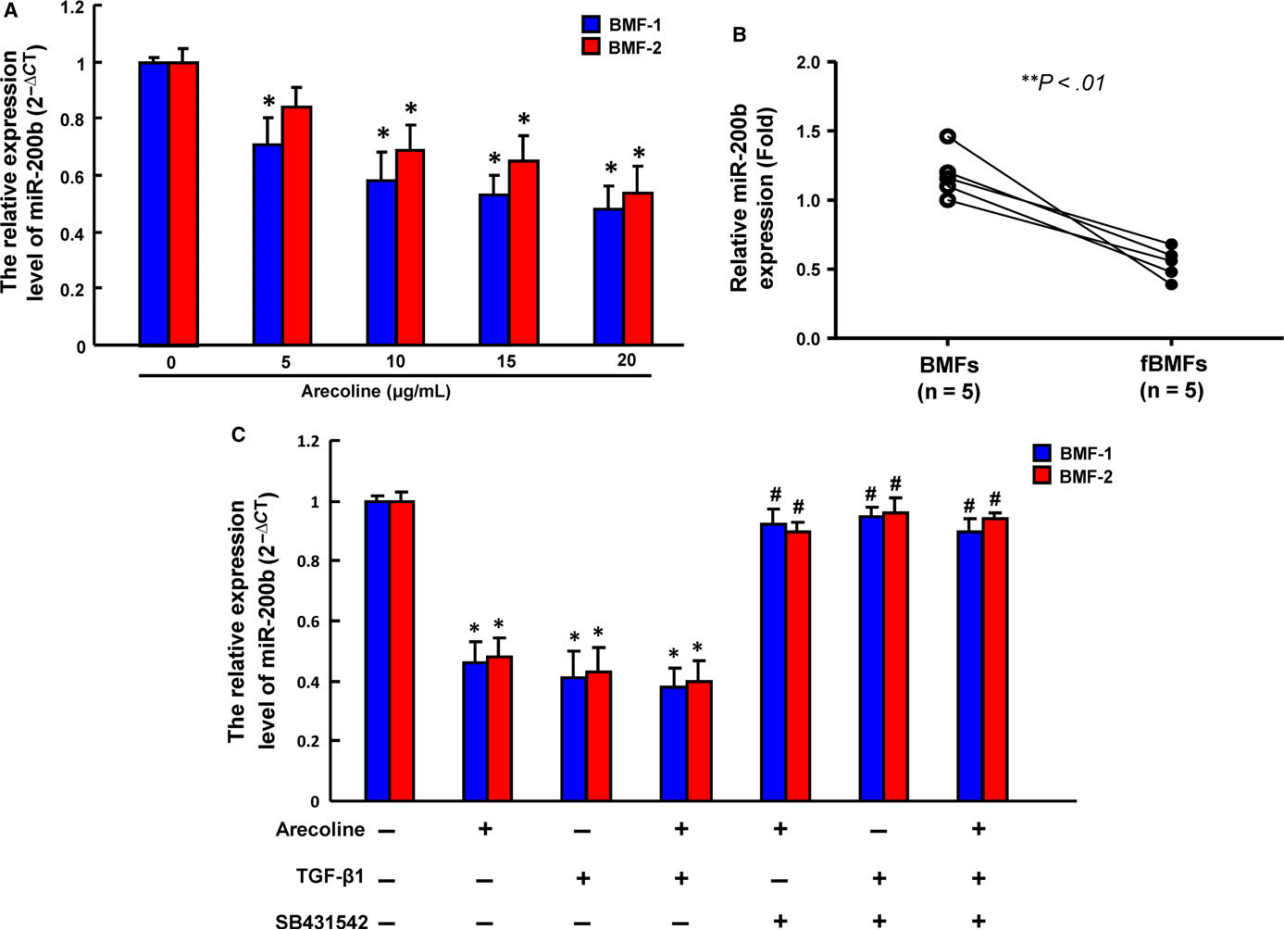
miR‐200b is down‐regulated in arecoline‐stimulated BMFs and fBMFs. The expression of miR‐200b was assessed in BMFs in response to various concentration of arecoline exposure (A); the expression of miR‐200b was compared between normal buccal mucosal fibroblasts (BMFs) and fibrotic BMFs performed with qRT‐PCR (B); C, cells were pretreated with TGF‐ß type I receptor inhibitor, SB431542 (10 μmol/L), followed by treatment with arecoline (20 mg/mL) or TGF‐ß1 (5 ng/mL) for 2 h. miR‐200b expression was measured using qRT‐PCR analysis. *P < .05 compared with no treatment control; **P < .01 compared with BMFs; ^#^ *P* < .05 compared to arecoline‐treated, TGF‐β1‐treated or combined‐treatment groups

Arecoline is a major areca nut alkaloid and has been implicated in the pathogenesis of OSF.^3^ Our previous study has demonstrated that arecoline could induce myofibroblast transdifferentiation in human primary buccal mucosal fibroblasts (BMFs).^13^ qPCR analysis revealed that the expression of miR‐200b reduced in both BMFs as the concentration of arecoline increased (Figure 1A). Likewise, primary cultivated fibroblasts from OSF tissues (fBMFs) displayed a significantly lower expression of miR‐200b in comparison with pair normal BMFs (Figure 1B). To examine whether the inhibition of miR‐200b by arecoline in BMFs was through TGF‐ß signalling, we pretreated the BMFs with SB431542 (10 μmol/L), TGF‐ß type I receptor inhibitor, followed by arecoline or TGF‐ß administration. As expected, SB431542 treatment significantly prevented the arecoline‐ or TGF‐ß1‐inhibited miR‐200b expression in BMFs (Figure 1C). These results showed that the alteration of miR‐200b after arecoline stimulation was via TGF‐ß signalling and may be associated with the OSF development; therefore, we conducted the following experiments to investigate the functional role of miR‐200b in myofibroblast characteristics.

### 3.2 Overexpression of miR‐200b successfully hinders the arecoline‐induced myofibroblast activities

**Figure 2:**
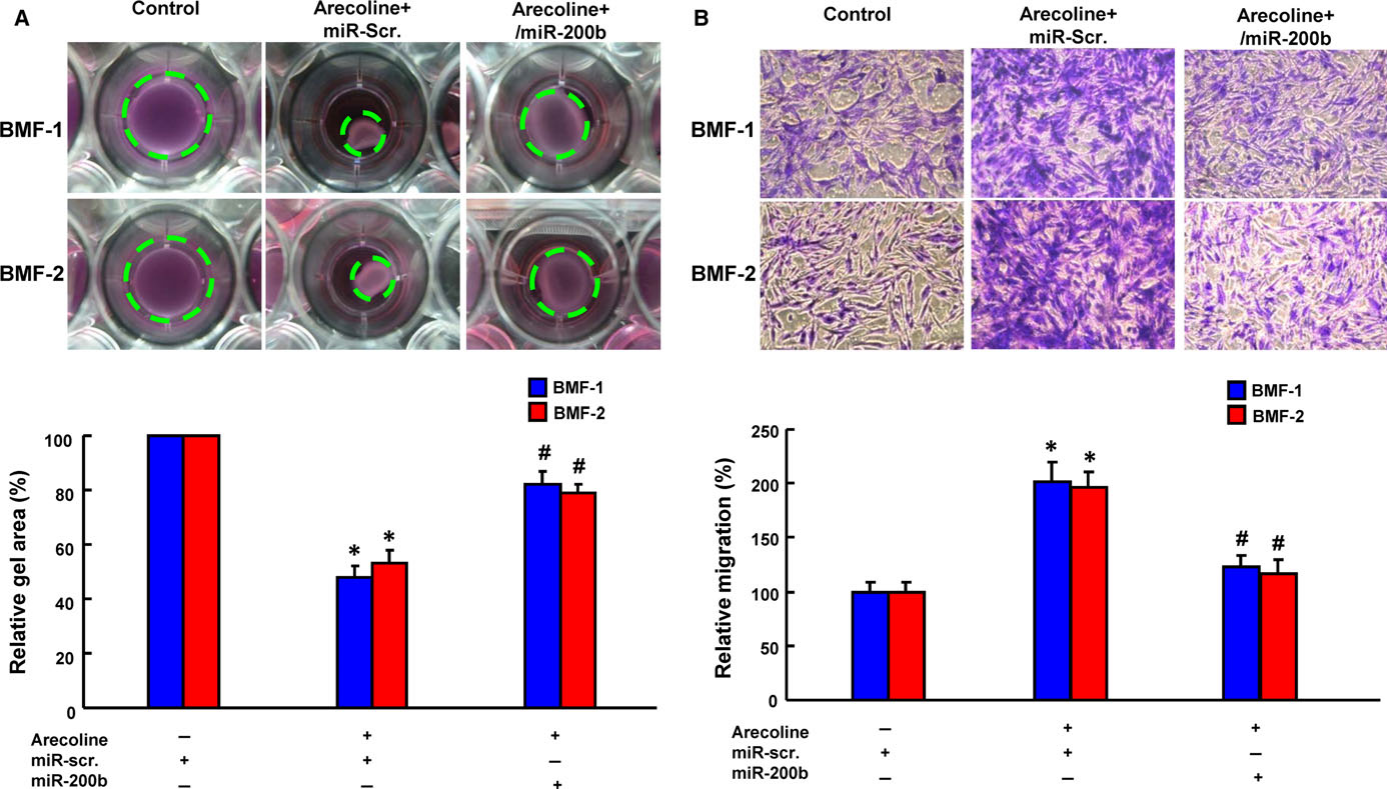
Arecoline‐induced myofibroblast activities are ameliorated by overexpression of miR‐200b. BMFs were treated with arecoline coordinately overexpression of miR‐200b followed by collagen gel contraction (A) and transwell migration (B) assays. *P <* *.05 compared with no treatment control; ^#^ *P *<* *.05 compared with arecoline + miR‐scr. group

Upon injury, fibroblasts become activated to migrate into the injured site and differentiate into contractile myofibroblasts for tissue healing.^6^ It also has been demonstrated that treatment of areca nut extract dose‐dependently increases the collagen contractility.^31^ Therefore, collagen gel contraction and migration capacities have been commonly employed to study the activity of myofibroblasts.^14^, ^29^ As expected, BMFs exhibited increased contractility in response to arecoline exposure, whereas overexpression of miR‐200b counteracted it (Figure 2A). In addition, we observed that the arecoline‐enhanced migration activity of BMFs was repressed by overexpression of miR‐200b (Figure 2B).

### 3.3 MiR‐200b reduces the characteristics of myofibroblasts

**Figure 3:**
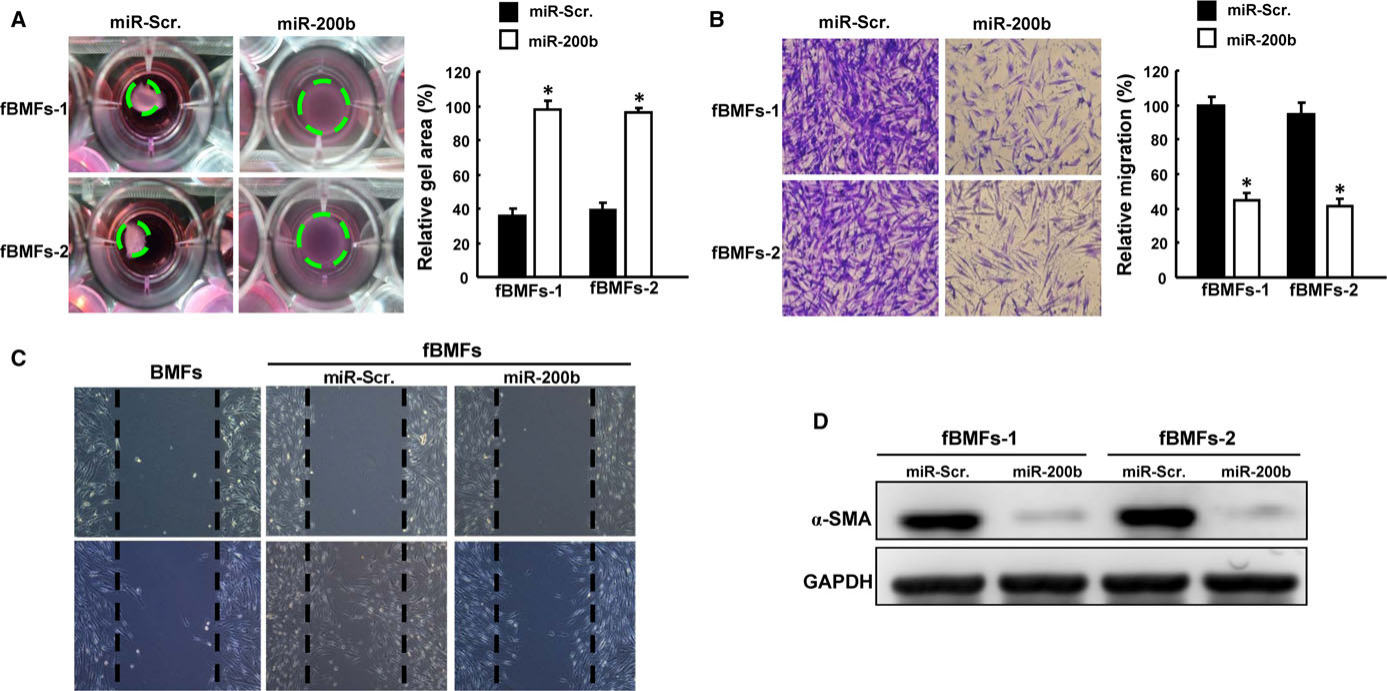
Overexpression of miR‐200b diminishes the elevated myofibroblast activities. Collagen gel contraction (A), transwell migration (B) and wound healing (C) assays were applied to examine the myofibroblast activities in two lines of patient‐derived fBMFs with or without overexpression of miR‐200b. (D) The expression of α‐SMA was evaluated by Western blot. *P <* *.05 compared with miR‐scr control

To confirm the role of miR‐200b in myofibroblast activation, we investigated whether these increased activities and myofibroblast marker, α‐SMA, would be suppressed by elevated expression of miR‐200b. By collagen contraction assay, we demonstrated that overexpression of miR‐200b significantly inhibited the highly contractile phenotype in BMFs from fibrotic oral cavity tissues (Figure 3A). With regard to the impact of miR‐200b on their migration capacity, we observed a decreased cell migration performed with transwell (Figure 3B) and wound healing (Figure 3C) assays. Moreover, we showed that miR‐200b reduced the expression level of α‐SMA, indicating the anti‐fibrotic effect of miR‐200b (Figures 3D and S1).

### 3.4 MiR‐200b directly targets ZEB2 and mediates ZEB2‐regulated expression of α‐SMA and vimentin in fBMFs

**Figure 4:**
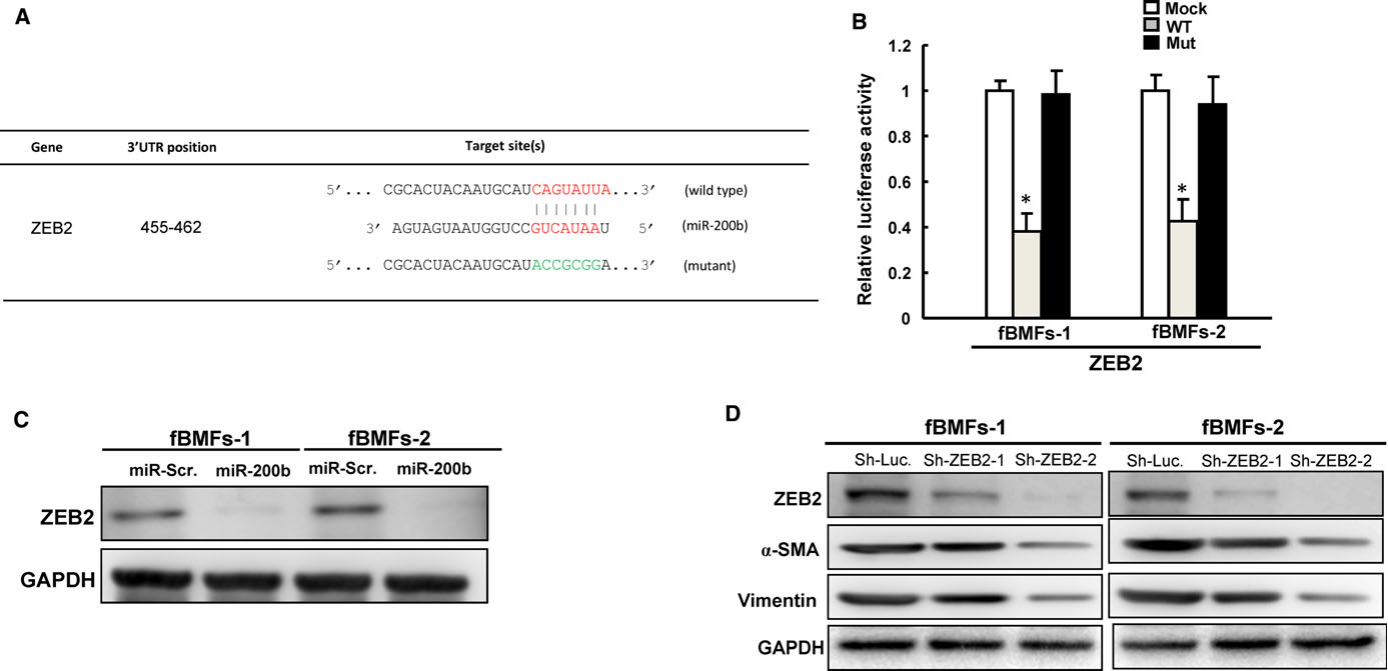
ZEB2 is a direct target of miR‐200b and knockdown of ZEB2 reduces the fibrosis markers. The illustration of 3′UTR regions of full‐length, mutated ZEB2 and miR‐200b (A); The luciferase activity of ZEB2 performed with mock, wild‐type and mutated construct was assessed (B); protein expression of ZEB2 with miR‐200b overexpression in fBMFs was detected by Western blot (C); protein expression of ZEB2, α‐SMA and vimentin was assessed following knockdown of ZEB2 (D). *P <* *.05 compared with mock control

As a direct target of miR‐200b,^32^, ^33^ ZEB2 (also known as SIP1) has been proved to induce α‐SMA and vimentin^34^ in cancer cells. Given that α‐SMA and vimentin are tightly associated with fibrosis,^35^, ^36^ we sought to assess the expression of ZEB2 and its effector proteins to elucidate the molecular mechanism underlying the reduced myofibroblast activities in the presence of miR‐200b. We illustrated complementarity between the 3′UTR regions of ZEB2 and miR‐200b (Figure 4A) to pinpoint the target sequence of miR‐200b in the 3′UTR of ZEB2. Reporter plasmids which contained either full‐length or mutated forms of ZEB2 were constructed and cotransfected with miR‐200b into fBMFs. Luciferase reporter assay demonstrated that miR‐200b reduced the luciferase activity of wild‐type ZEB2 but the mutated form was not affected (Figure 4B), indicating miR‐200b directly regulates ZEB2 in fBMFs. This result was verified by Western blot showing the expression of ZEB2 was reduced under overexpression of miR‐200 (Figures 4C and S2). Besides, we showed that knockdown of ZEB2 led to down‐regulation of α‐SMA and vimentin in fBMF (Figures 4D and S3).

### 3.5 Loss of miR‐200b stimulates the activation of BMFs and increases the expression of fibrosis markers

**Figure 5:**
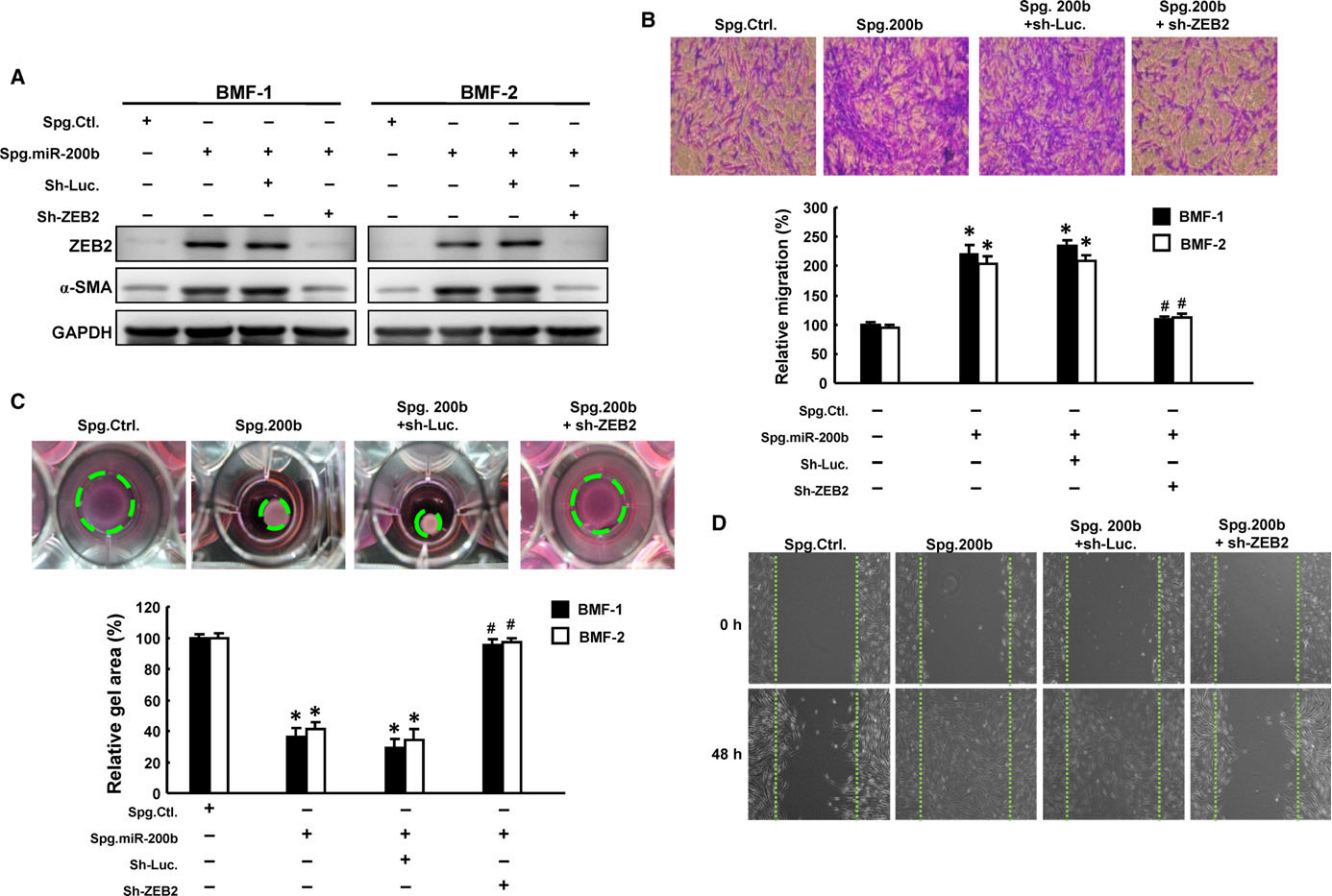
Loss of miR‐200b promotes myofibroblast differentiation through ZEB2. BMFs were transduced with miR‐200 sponge vector and/or shZEB2 lentivirus and examined for the expression of Slug and α‐SMA (A), transwell migration (B), collagen gel contraction (C) and wound healing (D) capacities. Contraction of the gels and migration ability were quantified using ImageJ software. *P <* *.05 compared with no treatment control; ^#^ *P *<* *.05 compared with Spg‐miR‐200b

To further confirm the functional involvement of miR‐200b in ZEB2‐mediated oral fibrosis, we treated BMFs with knockdown sponge (Spg. miR‐200b) and found the elevated expression of fibrosis‐associated markers, including Slug and α‐SMA, in comparison with cells treated with control sponge (Spg. Ctl.) (Figures 5A and S4). Nevertheless, the increased expression levels of Slug and α‐SMA were abolished by knockdown of ZEB2 (Figure 5A). We showed that silencing of endogenous miR‐200b induced higher migration ability (Figure 5B), collagen gel contractility (Figure 5C) and wound healing capacity (Figure 5D) in BMFs, whereas knockdown of ZEB2 counteracted these responses (Figure 5B‐D). Altogether, these findings suggested that blockage of the endogenous miR‐200b resulted in increased expression of Slug and α‐SMA via ZEB2 in BMFs, leading to increased myofibroblast activity.

### 3.6 Overexpression of miR‐200b expression induces apoptosis in fBMFs

Furthermore, we found that overexpression of miR‐200b increased the mean number of apoptotic cells in fBMFs (Figure S5A). miR‐200b suppressed the anti‐apoptotic genes Bcl‐2 and Bcl‐xl but elevated the Bax gene in fBMFs by real‐time RT‐PCR analysis (Figure S5B).

### 3.7 Reduced expression of miR‐200b and increased expression of ZEB2 in tissue specimens from OSF patients

**Figure 6:**
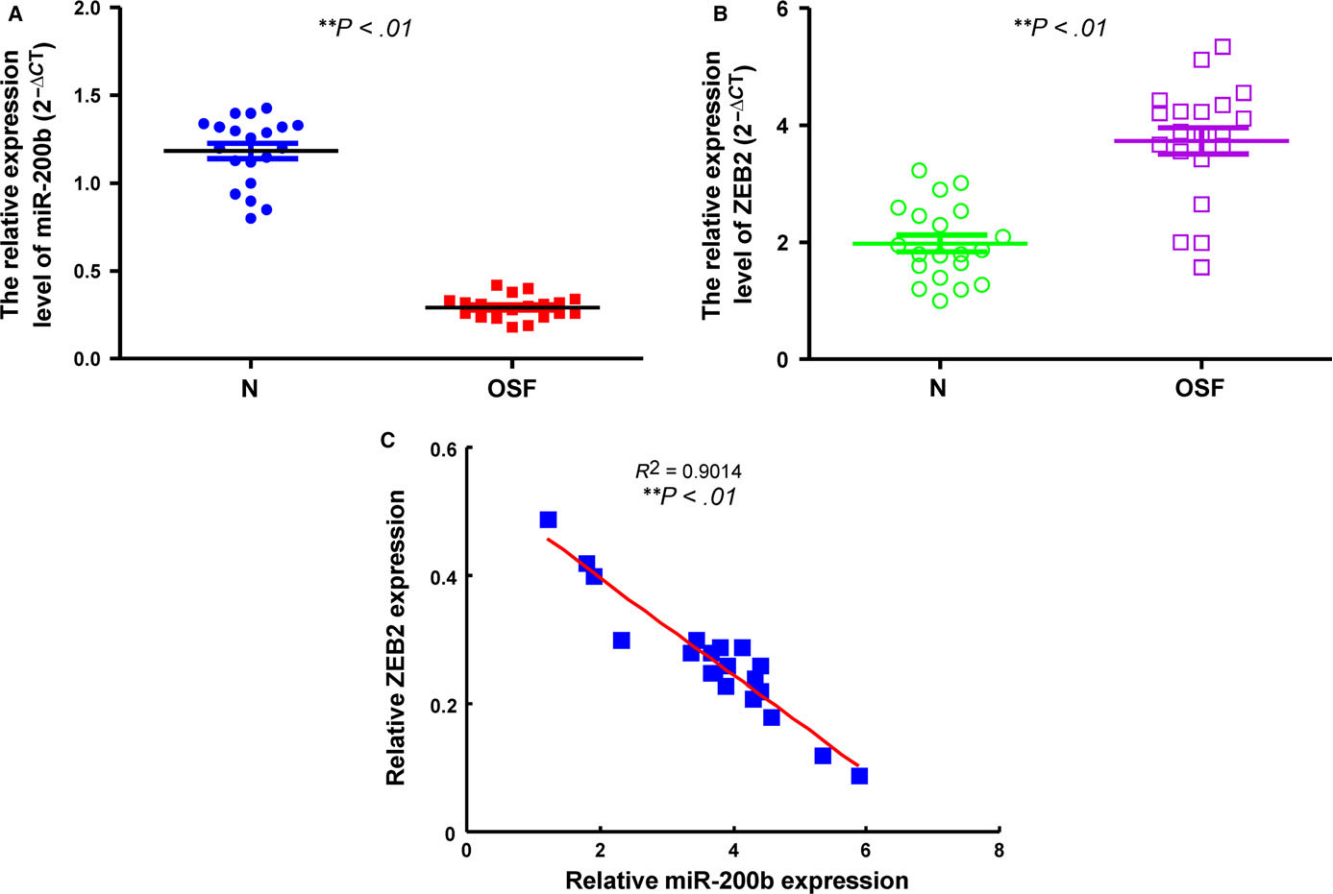
The expression of miR‐200b is down‐regulated and ZEB2 is up‐regulated in OSF tissues. The relative expression levels of miR‐200b (A) and ZEB2 (B) were detected and compared between normal (N) and OSF tissues **P <* *.01 compared with normal mucosal tissues. C, Total RNA was extracted from tissues of OSF patients and the expression of miR‐200b and ZEB2 was determined by qRT‐PCR methods and analysed with Spearman rank correlation test

To determine the clinical significance of miR‐200b and ZEB2, we examined the mRNA expression of miR‐200b and ZEB2 in OSF tissues. The expression level of miR‐200b was significantly lower in OSF specimens than normal group (Figure 6A). On the other hand, the relative gene expression of ZEB2 was up‐regulated in OSF tissues (Figure 6B). Linear regression analysis reveals a negative correlation between miR‐200b expression and ZEB2 mRNA expression in clinical OSF specimen (Figure 6C).

## 4 DISCUSSION

Despite emerging evidence has suggested that miR‐200 family members may involve in regulating multiple pathophysiological processes, the role of miR‐200b in the precancerous OSF has not been well characterized. In the current study, we demonstrated that miR‐200b is a critical factor in modulation of myofibroblast activity. Our results showed that overexpression of miR‐200b can prevent the elevated myofibroblast activity in BMFs after arecoline stimulation and we observed the similar effect in fBMFs with reduced expression of myofibroblast marker, α‐SMA, indicating that miR‐200b may possess the capacity to reverse myofibroblast transdifferentiation. Additionally, we proved that miR‐200b repressed Slug, vimentin and α‐SMA via directly binding to ZEB2, leading to diminished myofibroblast activity.

Over the past few years, the mechanism for the anti‐fibrotic effects of miR‐200b has been examined and discussed in several fibrotic diseases. It has been shown that miR‐200b ameliorated TGF‐β1‐induced fibrosis in colorectal epithelial cells.^37^ In hypertrophic scars, miR‐200b has been proved to decrease the expression of fibrosis markers, such as collagen type 1 and α‐SMA, as well as ZEB1 in fibroblasts.^38^ Another study showed that miR‐200b affected hypertrophic scarring through regulating the fibroblasts proliferation and apoptosis by affecting the collagen synthesis, fibronectin expression and TGF‐β1/α‐SMA signalling.^39^ As for pulmonary fibrosis, it has been found that the expression of miR‐200 was reduced in fibrotic lungs, and down‐regulation of miR‐200 may contribute to EMT and enhance pulmonary fibroblast accumulation.^22^ In contrast, overexpression of miR‐200b markedly attenuated TGF‐β1‐induced expression of fibronectin and α‐SMA in lung fibroblasts.^22^ Furthermore, overexpressing miR‐200b in intestinal epithelial cells led to inhibition of EMT characterized by down‐regulation of vimentin and up‐regulation of E‐cadherin through targeting ZEB1.^40^ One of the recent studies showed that miR‐200b/c exerts a protective effect in the LPS‐induced early pulmonary fibrosis by targeting ZEB1/2, which may be associated with the inhibition of p38 MAPK and TGF‐β/smad3 signalling.^41^ These data revealed that miR‐200b could regulate fibroblasts via reducing various fibrosis markers, such collagen type 1, fibronectin and α‐SMA. In consistent with these studies, we revealed the reduced expression of miR‐200b in OSF tissues and demonstrated that overexpression of miR‐200b reduced the hallmarks of myofibroblasts in arecoline‐stimulated BMFs and fBMFs.

Experimental data have identified various genes as the targets of miRNA‐200b. It has been indicated that miRNA‐200b regulated EMT via inhibition of ZEB1 and ZEB2.^23^, ^33^, ^42^, ^43^ ZEB2 is known as Smad‐interacting protein 1 (SIP1), and it has been shown that miRNA‐200b directly targets and represses Smad 2 in intestinal epithelial cells^40^ as well. One of the recent studies has discovered that level of miR‐200b was up‐regulated by miR‐192 in the mesangial cells, and miR‐192‐miR‐200 cascade induced TGF‐β1 expression.^44^ They showed that suppression of miR‐200b reversed the inhibitory effect of TGF‐β1 on ZEB1 protein expression and up‐regulated collagen via E‐box enhancer.^44^ Interestingly, another study reported that inhibition of miR‐192 caused elevated expression of ZEB1/2 and decreased collagen in diabetic renal fibrosis.^21^ It is worthy to examine the expression of miR‐192 in OSF tissues and investigate whether it affects the aberrant expression of miR‐200b in the future. In summary, this study demonstrated that miR‐200b regulated arecoline‐induced myofibroblast transdifferentiation by directly binding to ZEB2. We showed the anti‐fibrotic potential of miR‐200b in BMFs. Our results also provided evidence supporting that aberrant expression miR‐200b contributed to the pathogenesis of OSF and miR‐200b may function a screening factor and therapeutic target for OSF.

## CONFLICT OF INTEREST

The authors declare that they have no conflict of interests.

## References

[1] Velidandla S , Gaikwad P , Ealla KK , Bhorgonde KD , Hunsingi P , Kumar A . Histochemical analysis of polarizing colors of collagen using Picrosirius Red staining in oral submucous fibrosis. J Int Oral Health. 2014;6:33‐38. PMC3959134 24653600

[2] Isaac U , Issac JS , Ahmed Khoso N . Histopathologic features of oral submucous fibrosis: a study of 35 biopsy specimens. Oral Surg Oral Med Oral Pathol Oral Radiol Endod. 2008;106:556‐560. 18718788 10.1016/j.tripleo.2006.11.045

[3] Tilakaratne WM , Klinikowski MF , Saku T , Peters TJ , Warnakulasuriya S . Oral submucous fibrosis: review on aetiology and pathogenesis. Oral Oncol. 2006;42:561‐568. 16311067 10.1016/j.oraloncology.2005.08.005

[4] Chung CH , Yang YH , Wang TY , Shieh TY , Warnakulasuriya S . Oral precancerous disorders associated with areca quid chewing, smoking, and alcohol drinking in southern Taiwan. J Oral Pathol Med. 2005;34:460‐466. 16091112 10.1111/j.1600-0714.2005.00332.x

[5] Darby I , Skalli O , Gabbiani G . Alpha‐smooth muscle actin is transiently expressed by myofibroblasts during experimental wound healing. Lab Invest. 1990;63:21‐29. 2197503

[6] Hinz B , Phan SH , Thannickal VJ , Galli A , Bochaton‐Piallat ML , Gabbiani G . The Myofibroblast: one function, multiple origins. Am J Pathol. 2007;170:1807‐1816. 17525249 10.2353/ajpath.2007.070112 PMC1899462

[7] Wynn TA , Ramalingam TR . Mechanisms of fibrosis: therapeutic translation for fibrotic disease. Nat Med. 2012;18:1028‐1040. 22772564 10.1038/nm.2807 PMC3405917

[8] Angadi PV , Kale AD , Hallikerimath S . Evaluation of myofibroblasts in oral submucous fibrosis: correlation with disease severity. J Oral Pathol Med. 2011;40:208‐213. 21198872 10.1111/j.1600-0714.2010.00995.x

[9] LeBleu VS , Taduri G , O'Connell J , et al. Origin and function of myofibroblasts in kidney fibrosis. Nat Med. 2013;19:1047‐1053. 23817022 10.1038/nm.3218 PMC4067127

[10] Medici D , Hay ED , Olsen BR . Snail and Slug promote epithelial‐mesenchymal transition through beta‐catenin‐T‐cell factor‐4‐dependent expression of transforming growth factor‐beta3. Mol Biol Cell. 2008;19:4875‐4887. 18799618 10.1091/mbc.E08-05-0506 PMC2575183

[11] Lamouille S , Xu J , Derynck R . Molecular mechanisms of epithelial‐mesenchymal transition. Nat Rev Mol Cell Biol. 2014;15:178‐196. 24556840 10.1038/nrm3758 PMC4240281

[12] Mendez MG , Kojima S , Goldman RD . Vimentin induces changes in cell shape, motility, and adhesion during the epithelial to mesenchymal transition. FASEB J. 2010;24:1838‐1851. 20097873 10.1096/fj.09-151639 PMC2874471

[13] Chang YC , Tsai CH , Lai YL , et al. Arecoline‐induced myofibroblast transdifferentiation from human buccal mucosal fibroblasts is mediated by ZEB1. J Cell Mol Med. 2014;18:698‐708. 24400868 10.1111/jcmm.12219 PMC4000120

[14] Lee YH , Yang LC , Hu FW , Peng CY , Yu CH , Yu CC . Elevation of Twist expression by arecoline contributes to the pathogenesis of oral submucous fibrosis. J Formos Med Assoc. 2016;115:311‐317. 26088962 10.1016/j.jfma.2015.05.009

[15] Pillai RS , Bhattacharyya SN , Filipowicz W . Repression of protein synthesis by miRNAs: how many mechanisms? Trends Cell Biol. 2007;17:118‐126. 17197185 10.1016/j.tcb.2006.12.007

[16] Bartel DP . MicroRNAs: genomics, biogenesis, mechanism, and function. Cell. 2004;116:281‐297. 14744438 10.1016/s0092-8674(04)00045-5

[17] Jiang X , Tsitsiou E , Herrick SE , Lindsay MA . MicroRNAs and the regulation of fibrosis. FEBS J. 2010;277:2015‐2021. 20412055 10.1111/j.1742-4658.2010.07632.x PMC2963651

[18] Zhou Q , Yang M , Lan H , Yu X . miR‐30a negatively regulates TGF‐β1‐induced epithelial‐mesenchymal transition and peritoneal fibrosis by targeting Snai1. Am J Pathol. 2013;183:808‐819. 23831330 10.1016/j.ajpath.2013.05.019

[19] Yamada M , Kubo H , Ota C , et al. The increase of microRNA‐21 during lung fibrosis and its contribution to epithelial‐mesenchymal transition in pulmonary epithelial cells. Respir Res. 2013;14:95. 24063588 10.1186/1465-9921-14-95 PMC3849377

[20] Morizane R , Fujii S , Monkawa T , et al. miR‐34c attenuates epithelial‐mesenchymal transition and kidney fibrosis with ureteral obstruction. Sci Rep. 2014;4:4578. 24694752 10.1038/srep04578 PMC3974136

[21] Putta S , Lanting L , Sun G , Lawson G , Kato M , Natarajan R . Inhibiting microRNA‐192 ameliorates renal fibrosis in diabetic nephropathy. J Am Soc Nephrol. 2012;23:458‐469. 22223877 10.1681/ASN.2011050485 PMC3294315

[22] Yang S , Banerjee S , de Freitas A , et al. Participation of miR‐200 in pulmonary fibrosis. Am J Pathol. 2012;180:484‐493. 22189082 10.1016/j.ajpath.2011.10.005 PMC3349843

[23] Xiong M , Jiang L , Zhou Y , et al. The miR‐200 family regulates TGF‐β1‐induced renal tubular epithelial to mesenchymal transition through Smad pathway by targeting ZEB1 and ZEB2 expression. Am J Physiol Renal Physiol. 2012;302:F369‐F379. 22012804 10.1152/ajprenal.00268.2011

[24] Oba S , Kumano S , Suzuki E , et al. miR‐200b precursor can ameliorate renal tubulointerstitial fibrosis. PLoS ONE. 2010;5:e13614. 21049046 10.1371/journal.pone.0013614 PMC2963611

[25] Kurashige J , Mima K , Sawada G , et al. Epigenetic modulation and repression of miR‐200b by cancer‐associated fibroblasts contribute to cancer invasion and peritoneal dissemination in gastric cancer. Carcinogenesis. 2015;36:133‐141. 25411357 10.1093/carcin/bgu232

[26] Pan B , Feng B , Chen Y , et al. MiR‐200b regulates autophagy associated with chemoresistance in human lung adenocarcinoma. Oncotarget. 2015;6:32805‐32820. 26416454 10.18632/oncotarget.5352 PMC4741731

[27] Ye F , Tang H , Liu Q , et al. miR‐200b as a prognostic factor in breast cancer targets multiple members of RAB family. J Transl Med. 2014;12:17. 24447584 10.1186/1479-5876-12-17 PMC3898994

[28] Yang SF , Tsai CH , Chang YC . The upregulation of heat shock protein 47 expression in human buccal fibroblasts stimulated with arecoline. J Oral Pathol Med. 2008;37:206‐210. 18221324 10.1111/j.1600-0714.2007.00633.x

[29] Yu CC , Yu CH , Chang YC . Aberrant SSEA‐4 upregulation mediates myofibroblast activity to promote pre‐cancerous oral submucous fibrosis. Sci Rep. 2016;6:37004. 27845370 10.1038/srep37004 PMC5109465

[30] Yang PY , Hsieh PL , Wang TH , et al. Andrographolide impedes cancer stemness and enhances radio‐sensitivity in oral carcinomas via miR‐218 activation. Oncotarget. 2017;8:4196‐4207. 27926533 10.18632/oncotarget.13755 PMC5354823

[31] Chang MC , Lin LD , Wu HL , et al. Areca nut‐induced buccal mucosa fibroblast contraction and its signaling: a potential role in oral submucous fibrosis–a precancer condition. Carcinogenesis. 2013;34:1096‐1104. 23349021 10.1093/carcin/bgt012

[32] Korpal M , Lee ES , Hu G , Kang Y . The miR‐200 family inhibits epithelial‐mesenchymal transition and cancer cell migration by direct targeting of E‐cadherin transcriptional repressors ZEB1 and ZEB2. J Biol Chem. 2008;283:14910‐14914. 18411277 10.1074/jbc.C800074200 PMC3258899

[33] Park SM , Gaur AB , Lengyel E , Peter ME . The miR‐200 family determines the epithelial phenotype of cancer cells by targeting the E‐cadherin repressors ZEB1 and ZEB2. Genes Dev. 2008;22:894‐907. 18381893 10.1101/gad.1640608 PMC2279201

[34] Nam EH , Lee Y , Park YK , Lee JW , Kim S . ZEB2 upregulates integrin α5 expression through cooperation with Sp1 to induce invasion during epithelial–mesenchymal transition of human cancer cells. Carcinogenesis. 2012;33:563‐571. 22227038 10.1093/carcin/bgs005

[35] Hinz B , Celetta G , Tomasek JJ , Gabbiani G , Chaponnier C . Alpha‐smooth muscle actin expression upregulates fibroblast contractile activity. Mol Biol Cell. 2001;12:2730‐2741. 11553712 10.1091/mbc.12.9.2730 PMC59708

[36] Yu CC , Tsai CH , Hsu HI , Chang YC . Elevation of S100A4 expression in buccal mucosal fibroblasts by arecoline: involvement in the pathogenesis of oral submucous fibrosis. PLoS ONE. 2013;8:e55122. 23383075 10.1371/journal.pone.0055122 PMC3561403

[37] Chen Y , Ge W , Xu L , et al. miR‐200b is involved in intestinal fibrosis of Crohn's disease. Int J Mol Med. 2012;29:601‐606. 22294131 10.3892/ijmm.2012.894 PMC3573760

[38] Zhou R , Zhang Q , Zhang Y , Fu S , Wang C . Aberrant miR‐21 and miR‐200b expression and its pro‐fibrotic potential in hypertrophic scars. Exp Cell Res. 2015;339:360‐366. 26500110 10.1016/j.yexcr.2015.10.018

[39] Li P , He QY , Luo CQ . Overexpression of miR‐200b inhibits the cell proliferation and promotes apoptosis of human hypertrophic scar fibroblasts in vitro. J Dermatol. 2014;41:903‐911. 25228082 10.1111/1346-8138.12600

[40] Chen Y , Xiao Y , Ge W , et al. miR‐200b inhibits TGF‐β1‐induced epithelial‐mesenchymal transition and promotes growth of intestinal epithelial cells. Cell Death Dis. 2013;4:e541. 23492772 10.1038/cddis.2013.22 PMC3613822

[41] Cao Y , Liu Y , Ping F , Yi L , Zeng Z , Li Y . miR‐200b/c attenuates lipopolysaccharide‐induced early pulmonary fibrosis by targeting ZEB1/2 via p38 MAPK and TGF‐beta/smad3 signaling pathways. Lab Invest. 2017;98:339‐359. 29200203 10.1038/labinvest.2017.123

[42] Tang O , Chen XM , Shen S , Hahn M , Pollock CA . MiRNA‐200b represses transforming growth factor‐β1‐induced EMT and fibronectin expression in kidney proximal tubular cells. Am J Physiol Renal Physiol. 2013;304:F1266‐F1273. 23408168 10.1152/ajprenal.00302.2012

[43] Gregory PA , Bert AG , Paterson EL , et al. The miR‐200 family and miR‐205 regulate epithelial to mesenchymal transition by targeting ZEB1 and SIP1. Nat Cell Biol. 2008;10:593‐601. 18376396 10.1038/ncb1722

[44] Kato M , Arce L , Wang M , Putta S , Lanting L , Natarajan R . A microRNA circuit mediates transforming growth factor‐β1 autoregulation in renal glomerular mesangial cells. Kidney Int. 2011;80:358‐368. 21389977 10.1038/ki.2011.43 PMC3337779

