# Supplementary figures and images for: miR‐200b ameliorates myofibroblast transdifferentiation in precancerous oral submucous fibrosis through targeting ZEB2
Source: J Cell Mol Med. 2018 Jun 12;22(9):4130–8. doi: 10.1111/jcmm.13690 (PMC6111815; doi:10.1111/jcmm.13690)

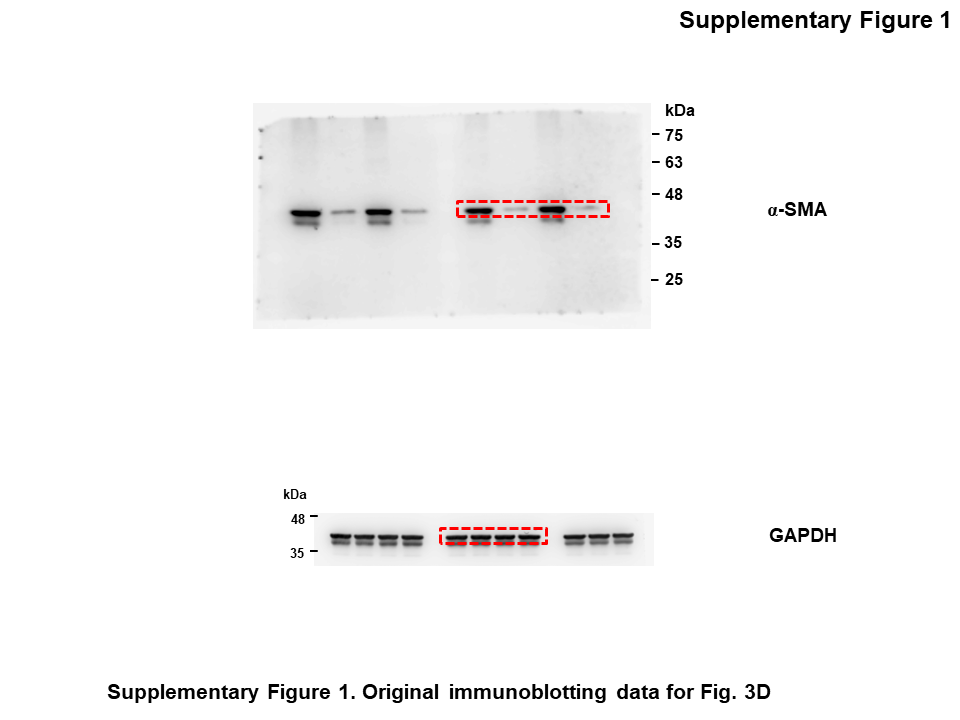

Supplement: Supplementary file 1 [file JCMM-22-4130-s001.TIF]

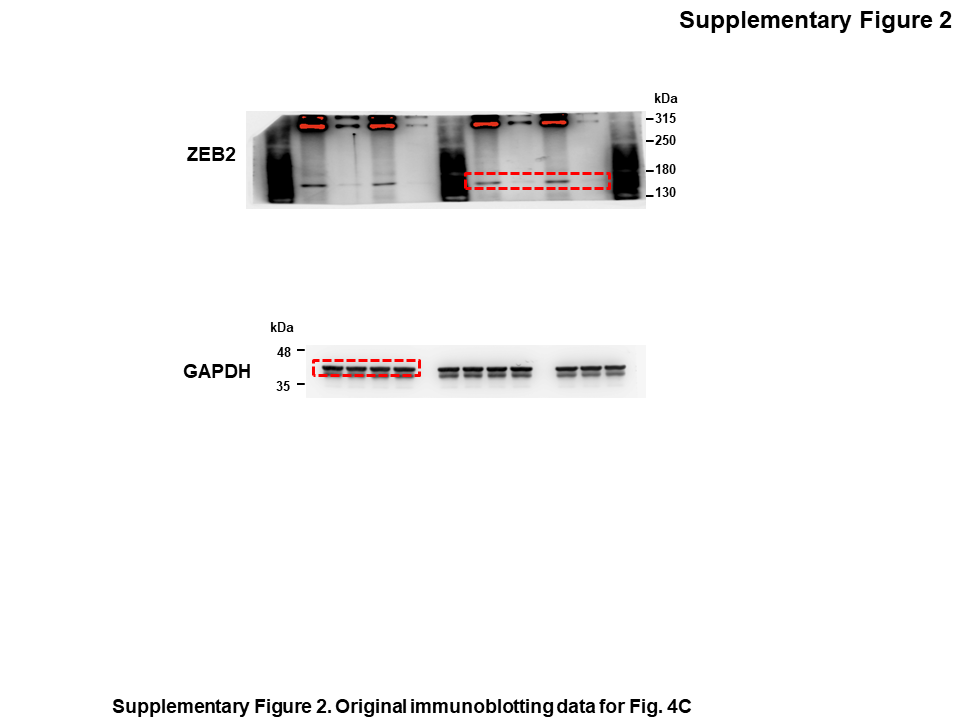

Supplement: Supplementary file 2 [file JCMM-22-4130-s002.TIF]

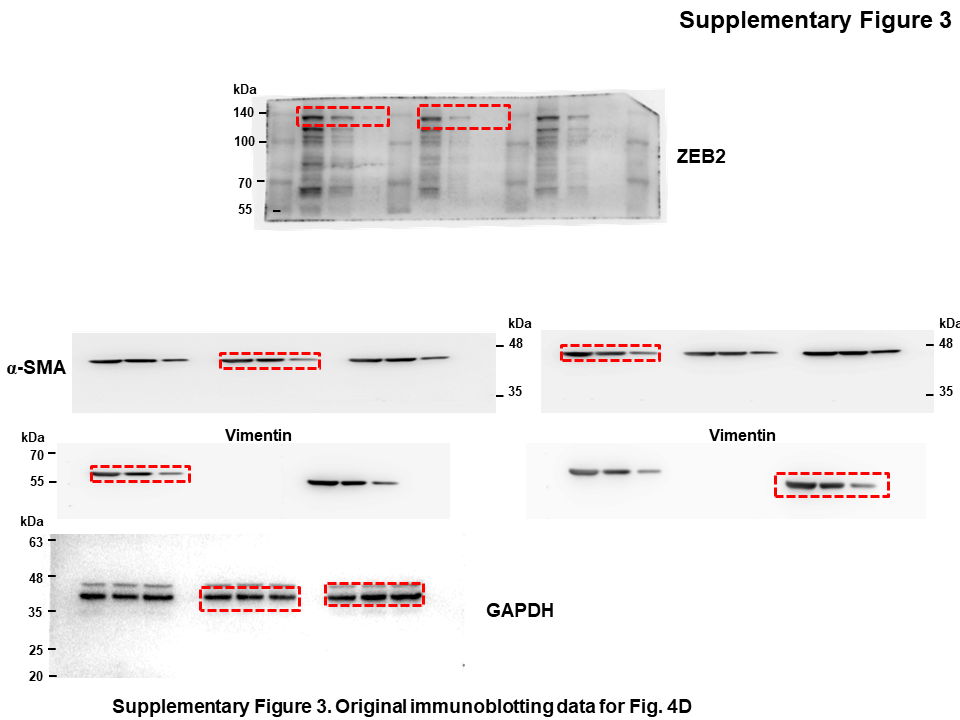

Supplement: Supplementary file 3 [file JCMM-22-4130-s003.TIF]

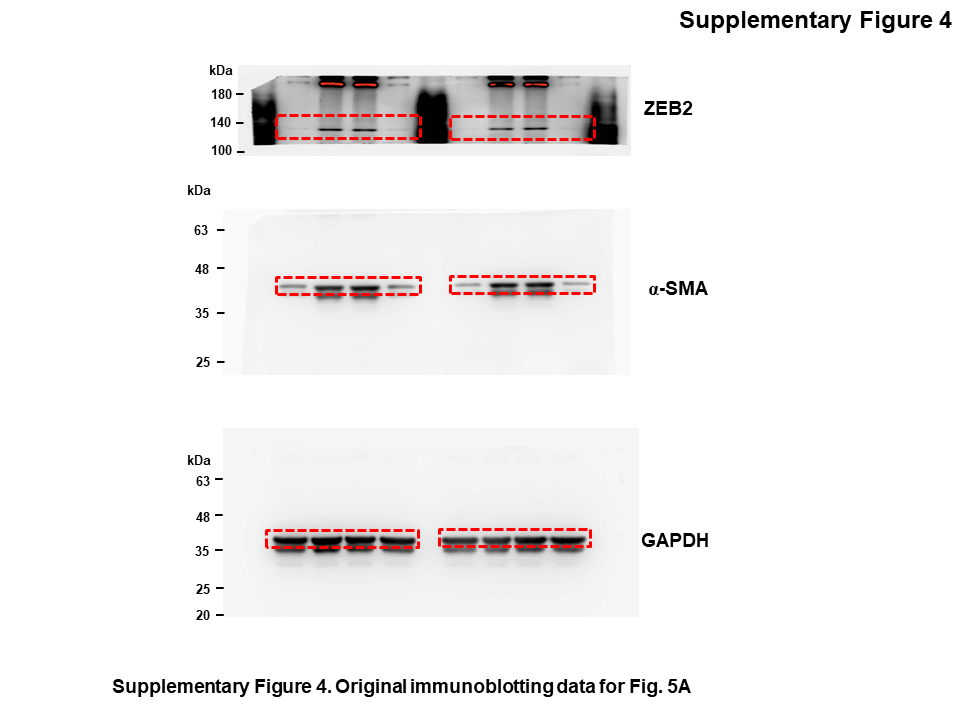

Supplement: Supplementary file 4 [file JCMM-22-4130-s004.TIF]

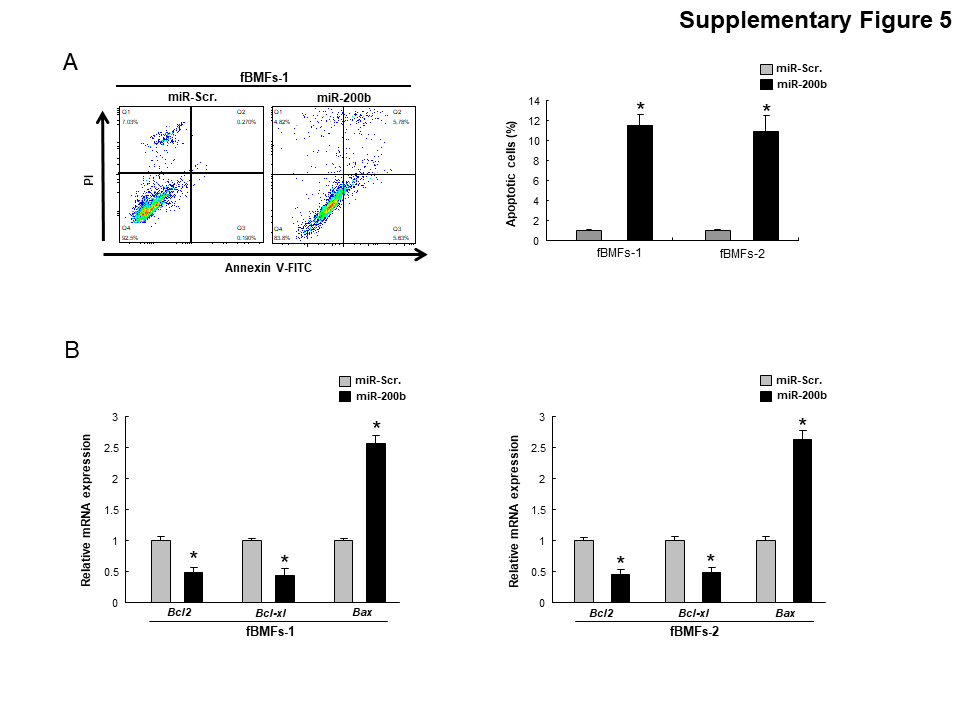

Supplement: Supplementary file 5 [file JCMM-22-4130-s005.TIF]
