# Supplementary material for: miR‐200b ameliorates myofibroblast transdifferentiation in precancerous oral submucous fibrosis through targeting ZEB2
Source: J Cell Mol Med. 2018 Jun 12;22(9):4130–8. doi: 10.1111/jcmm.13690 (PMC6111815; doi:10.1111/jcmm.13690)
Supplement: Supplementary file 6 [file JCMM-22-4130-s006.docx]

Supplementary information

**Supplementary Figure 5. miR-200b induces apoptosis in fBMFs**

(A) Annexin V -positive apoptosis cells were assessed in fBMFs treated with miR-Scr. and miR-200b. (F) The expression levels of the Bcl2, Bcl-xl and Bax in the cells indicated were determined by quantitative RT-PCR. * *p* < 0.05 compared with control.
